# Combined effect of thymectomy on myasthenia gravis in patients with concomitant auto-immune diseases: a 22-year single-center experience

**DOI:** 10.1007/s13304-023-01568-7

**Published:** 2023-06-27

**Authors:** Lei Liu, Jiaqi Zhang, Chao Guo, Yeye Chen, Hongsheng Liu, Shanqing Li, Cheng Huang

**Affiliations:** Department of Thoracic Surgery, Peking Union Medical College Hospital, Chinese Academy of Medical Sciences and Peking Union Medical College, Peking, No.1 Shuaifuyuan, Wangfujing Street, Dongcheng District, Beijing, 100730 People’s Republic of China

**Keywords:** Myasthenia gravis, Autoimmune disease, Thymectomy, Thymomectomy, Prognosis

## Abstract

Myasthenia gravis (MG) is an autoimmune disease (AD), and patients with MG often have other types of ADs. We analyzed the prognosis of patients with MG complicated by AD after thymectomy. A retrospective analysis was performed for patients with MG complicated by ADs treated surgically in our center over the past 22 years, and their general condition and follow-up data were collected and analyzed. 33 patients were included totally. 28 patients displayed improvement or even complete recovery of MG, and 23 of 36 ADs revealed improvement or even complete recovery. The prognosis of MG is significantly correlated with the duration of postoperative follow-up time (*p* = 0.028), and in patients with thymoma, the larger the tumor diameter, the better the prognosis of MG (*p* = 0.026). Thymic hyperplasia patients were predominantly female (*p* = 0.049) and young (*p* < 0.001). The most common concomitant AD in this study was a thyroid-associated AD, which was associated with thymic hyperplasia (*p* < 0.001), Osserman type I MG (*p* < 0.001), and young age (*p* < 0.001). Thymectomy had a good therapeutic effect on MG complicated by AD, and there was a close correlation between surgery, thymus, MG, and ADs.

## Introduction

Myasthenia gravis (MG) is an autoimmune disease (AD) caused by specific autoantibodies in the postsynaptic membrane of neuromuscular junctions. MG patients have a 22% risk of other ADs during their lifetime compared to 9% in the MG-free general population [1]. Previous research depicts MG has been linked to numerous ADs, including autoimmune thyroid disease, systemic lupus erythematosus, and rheumatic arthritis [2].

Thymectomy or thymomectomy is one of the most effective ways to treat MG [3, 4]. The results of available studies on MG remission after surgery in MG patients accompanied by autoimmune disease are inconsistent [5, 6]. Meanwhile, there have been some studies on AD prognosis after thymectomy in MG patients [7–9].

Our team's previous studies have found that surgical treatment of patients with thymoma accompanied by non-MG AD effectively relieves their condition [10]. Therefore, we were interested in the postoperative outcomes of thymectomy/thymomectomy in MG patients accompanied by AD. Considering that no studies combined MG and AD in the analysis, we designed this study.

## Methods

We reviewed 460 patients who received MG treatment at Peking Union Medical College Hospital from 2000 to 2022. The inclusion criteria were MG with at least one AD and surgical treatment (thymectomy/thymomectomy). Patients were divided into thymoma and non-thymoma groups according to the presence or absence of thymoma. Preoperative MG was classified according to the Osserman classification [11]. The patients were followed up by telephone or during outpatient visits. The data collected from the follow-ups included the treatment of MG and AD before and after the operation, recurrence, and metastasis of the tumor after the operation, and prognosis of MG and AD. The improvement in MG and AD was reflected by the continuous decrease in immune-related indices, improved clinical symptoms, recovery of laboratory test indices related to ADs, and decreased or withdrawal of the drug dosage needed to alleviate the respective symptoms. After follow-up, sex, age, MG subtype, operation mode, postoperative pathology, and prognosis of MG and AD were analyzed and recorded. This retrospective study was performed under authorization approved by the Institutional Review Board of Peking Union Medical College Hospital, Beijing, China (No.K2181).

We categorized the MG postoperative status into the following classifications: complete stable remission (remission without Prednisone), pharmacological remission (remission with medium/low dose Prednisone), stable, and depraved. In this study, we defined complete stable remission (remission without Prednisone) and pharmacological remission (remission with medium/low dose Prednisone) as improved.

SPSS software (version 26.0) was used to perform rank correlation analysis. Pearson's correlation and Mann–Whitney *U* tests were used to analyzing the correlation between groups. Clinical significance was set at *p* < 0.05.

## Results

### General situation of the included population

In total, 33 patients were included in this study, including 11 males and 22 females, with a median age of 38 (15–76) years. Regarding the pathological diagnosis of the enrolled patients, 16 were diagnosed with simple thymic hyperplasia and 17 with thymoma. Two surgical approaches were used: median thoracotomy and video-assisted thoracoscopic surgery (VATS). There were no major perioperative complications or deaths in this group of patients. The median follow-up period in this review was 95 (5–235) months. At the last follow-up, only one patient died of septic shock nearly ten years postoperatively, and no recurrences or metastases existed in patients with a pathological diagnosis of thymoma.

### Conditions of MG and AD

Among the 33 patients included, MG's Osserman classification was distributed from I to IIB, and different doses and frequencies of pyridostigmine bromide were used preoperatively to control symptoms. Some patients were also treated with a combination of hormones (prednisone/methylprednisolone/prednisone) before surgery. There were 30 patients with one AD and 3 with two ADs. Among the comorbid ADs, thyroid-related AD (TRAD) was the most common, with 17 patients having predominantly concomitant AD as TRAD, including Graves' disease, hyperthyroidism, hypothyroidism, and Hashimoto's thyroiditis. Besides TRAD, the other types of immune diseases included irritant dermatitis, pure red cell aplasia, allergic rhinitis, Henoch–Schönlein purpura, glomerulonephritis, connective tissue disease, asthma, primary biliary cirrhosis, and urticaria (Table 1).

**Table 1:** General conditions of included patients and prognosis of MG and AD

No	Age/gender	Pathology	Masaoka stage	Osserman type	Prognosis	AD1	Prognosis	AD2	Prognosis
1	44/M	B2 Thymoma	III	IIB	Improved	CTD	Improved	–	–
2	50/M	B1 Thymoma	I	I	Improved	Allergic rhinitis	Improved	–	–
3	46/M	B1 Thymoma	III	IIA	Improved	PRCA	Improved	–	–
4	76/M	B1 Thymoma	I	I	Improved	Graves’ disease	Improved	–	–
5	65/M	B1 Thymoma	I	I	Improved	Urticaria	Improved	–	–
6	38/M	B2 Thymoma	II	IIB	Improved	Glomerulonephritis	Improved	–	–
7	43/M	B1 Thymoma	I	IIB	Depraved	Irritant dermatitis	Improved	–	–
8	24/M	TH	NA	IIB	Improved	Hyperthyroidism	Stable	–	–
9	36/M	B2 Thymoma	II	I	Improved	Allergic rhinitis	Stable	–	–
10	61/M	B2 + B3Thymoma	III	IIA	Improved	Hypothyroidism	Stable	–	–
11	35/M	AB Thymoma	I	IIB	Improved	Asthma	Stable	–	–
12	27/F	TH	NA	I	Improved	Hashimoto's thyroiditis	Improved	–	–
13	16/F	TH	NA	I	Improved	Hyperthyroidism	Improved	–	–
14	18/F	TH	NA	I	Stable	Hyperthyroidism	Improved	–	–
15	25/F	TH	NA	I	Improved	Hyperthyroidism	Improved	–	–
16	15/F	TH	NA	I	Improved	Hyperthyroidism	Improved	–	–
17	22/F	TH	NA	IIA	Improved	HSP	Improved	–	–
18	45/F	TH	NA	I	Stable	Graves’ disease	Improved	–	–
19	26/F	TH	NA	I	Improved	Hyperthyroidism	Improved	–	–
20	26/F	TH	NA	I	Improved	Hyperthyroidism	Improved	–	–
21	53/F	TH	NA	IIA	Improved	Hypothyroidism	Improved	CTD	Stable
22	37/F	TH	NA	I	Improved	Hyperthyroidism	Improved	Urticaria	Improved
23	31/F	B1 + B2 Thymoma	I	IIB	Improved	Hashimoto's thyroiditis	Improved	CTD	Stable
24	52/F	B1 Thymoma	I	IIA	Improved	PBC	Improved	–	–
25	68/F	B2 Thymoma	I	IIB	Improved	Asthma	Improved	–	–
26	46/F	B2 Thymoma	II	I	Improved	Allergic rhinitis	Improved	–	–
27	46/F	B1 Thymoma	II	IIB	Improved	Hypothyroidism	Stable	–	–
28	24/F	TH	NA	IIB	Improved	Hyperthyroidism	Stable	–	–
29	25/F	B2 Thymoma	II	I	Improved	Asthma	Stable	–	–
30	49/F	AB Thymoma	II	IIB	Improved	Asthma	Stable	–	–
31	38/F	TH	NA	I	Improved	Hyperthyroidism	Stable	–	–
32	19/F	TH	NA	I	Stable	Hyperthyroidism	Depraved	–	–
33	49/F	B1 Thymoma	I	IIA	Depraved	Asthma	Depraved	–	–

*AD* autoimmune disease, *TH* thymic hyperplasia, *PRCA* pure red cell aplasia, *CTD* connective tissue disease, *PBC* primary biliary cirrhosis and urticarial, *HSP* Henoch–Schönlein purpura

At the last follow-up, 28 MG patients demonstrated complete or partial improvement, with an improvement rate of 84.85%; 3 with MG had no obvious postoperative symptom changes, and 2 had worsening MG symptoms after surgery. In terms of AD, of the 36 ADs, 23 demonstrated complete or partial improvement, 1 depicted insignificant change compared to the preoperative state, and 2 depicted deterioration. Concerning the major combined ADs, 22 patients demonstrated improvement, with an improvement rate of 66.67%; 9 demonstrated insignificant change, and 2 deteriorated.

### Data analysis

**Fig. 1:**
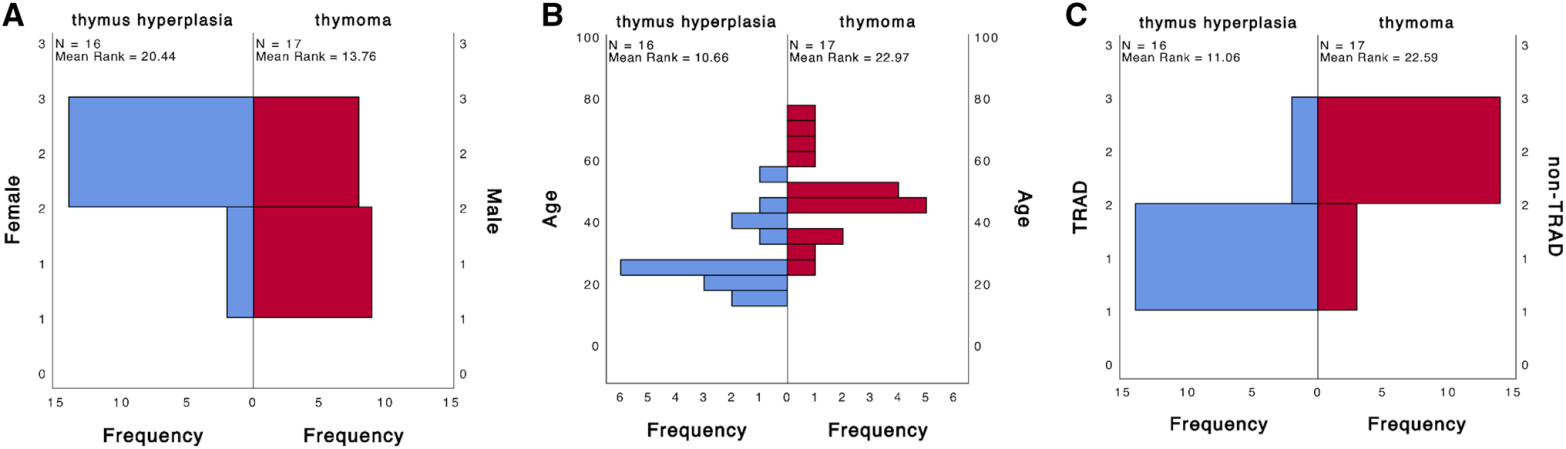
Distribution differences between thymus hyperplasia and thymoma: **A** gender; **B** age; **C** type of autoimmune disease. *TRAD* thyroid-related autoimmune disease

**Fig. 2:**
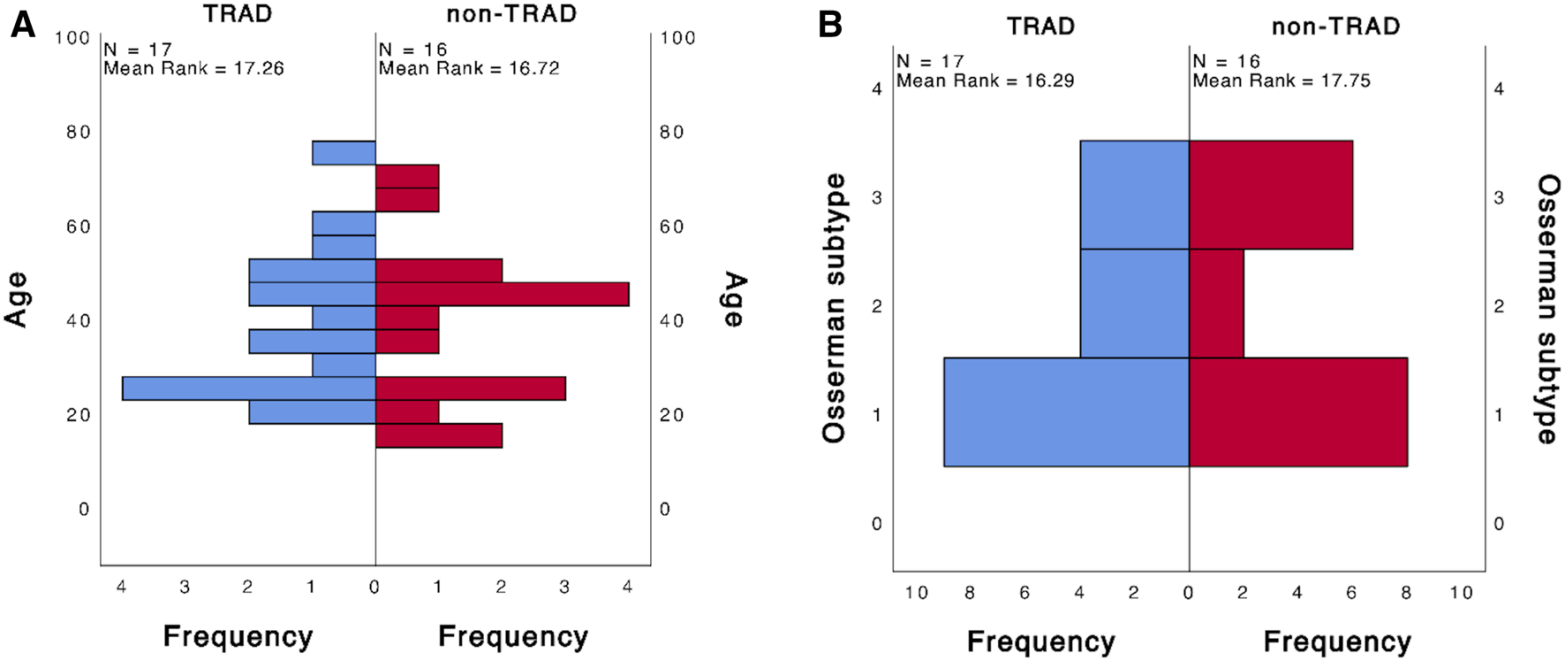
Distribution differences between different types of autoimmune disease: **A** age; **B** Osserman subtype. *TRAD* thyroid-related autoimmune disease

Using Pearson's correlation, we found that the improvement after MG was proportional to the postoperative follow-up time, and the longer the postoperative period, the better the resolution of MG (Pearson's correlation coefficient = 0.384, *p* = 0.028). However, this feature was not observed in the prognosis of autoimmune diseases (Pearson's correlation coefficient = 0.329, *p* = 0.062). In addition, we found that patients with thymoma had significantly greater postoperative muscle weakness relief (Pearson's correlation coefficient = 0.509, *p* = 0.026). In addition, we found that patients with larger thymoma had significantly greater postoperative MG relief (Pearson's correlation coefficient = 0.509, *p* = 0.026). Using the Mann–Whitney U test, we found that among the enrolled patients, thymic hyperplasia was correlated with female gender (*p* = 0.049), younger age (*p* < 0.001), and TRAD (*p* < 0.001) (Fig. 1). In addition, we found that TRAD patients were younger (*p* = 0.049) and associated with Osserman's type (*p* = 0.049) (Fig. 2).

## Discussion

To the best of our knowledge, there are no generally agreed-upon definitions of AD. Davidson et al*.* defined AD as "a clinical syndrome caused by the activation of T cells, B cells, or both in the absence of an ongoing infection or other discernible causes" [12]. MG is currently considered to be an AD with multiple clinical manifestations. After the first case of Graves' disease with thymic hyperplasia and the first case of thymoma resection for MG [13, 14], research on thymectomy/thymomectomy, MG, and AD has gradually flourished. We included MG patients who had undergone surgery and were diagnosed with AD. To the best of our knowledge, this is the first study on postoperative outcomes in patients with both MG and AD.

The thymus plays a central role in maintaining immune tolerance toward self-antigens, which is considered the initial checkpoint in preventing autoimmunity [15, 16]. Lymphoepithelioma in the thymus increases the risk of a few rare autoimmune disorders, including blood cytopenia, hypogammaglobulinemia, polymyositis, and neuromyotonia [17]. ADs were diagnosed in 38.8% of thymoma patients, while they were significantly less common in both thymic carcinomas and neuroendocrine thymic tumors (5.8% and 3.6%, respectively). Among all ADs, MG was the most prominent, with an incidence of 34%. Pure red cell aplasia, hypogammaglobulinemia, and a mixture of other ADs account for less than 1% of all cases [18]. Thymic hyperplasia is a marker of an increased risk of AD [9].

This study focused on MG and AD outcomes after surgery. Among the enrolled patients, 84.85% achieved complete or partial improvement in MG symptoms after surgery, and 66.67% of AD patients exhibited complete or partial improvement. Previous studies have confirmed that surgical removal of the thymus/thymoma is meaningful for relieving MG [19–21]. In addition, we found that in patients with thymoma, the larger the tumor diameter, the better the postoperative prognosis of MG. Previous studies have reported that patients with thymoma in the early Masaoka stage have better postoperative MG remission [22]. Although tumor diameter was not associated with the Masaoka stage, we found contradictions in the above study. We believe that the larger the thymoma, the greater the potential to cause immune dysfunction and the stronger the dominant force for the development of MG. Therefore, the relief of MG after thymoma removal is also more pronounced. We found that MG symptoms improved over time after surgery. Unfortunately, this study did not find a correlation between the follow-up time after surgery and AD outcome, possibly because the pathogenesis of non-MG AD is related to the complex systemic immune system, not just the thymus. However, we also found that the response rate for AD is approximately 70%. Previous studies have also found that the response after thymectomy for MG accompanied by AD is comparable to the response of most MG patients, but thymectomy does not influence hyperthyroidism [23].

After dividing the enrolled patients according to their pathological types into thymic hyperplasia and thymoma groups, we found some differences between the two groups. This study found that thymic hyperplasia was more common in younger people and women. This was consistent with the clinical features of early-onset MG reported in a previous study [24]. Recently, some studies have also reported that MG patients with thymoma are younger, but no difference in sex distribution has been reported [25]. Simultaneously, we found that patients with thymic hyperplasia were more likely to have concomitant TRAD. In 1912, Scheiff et al*.* reported a case of thymic hyperplasia and Graves' disease [13]. Recent studies have found that removing a malfunctioning thymus reduces the prevalence of positive antithyroid autoantibodies in MG patients [7]. Therefore, thymic hyperplasia might play a role in TRAD occurrence and development.

Consistent with previous findings [2, 26], the most common co-occurring AD in our cohort was TRAD. Previous research has reported that chaotic expression of the thyroid-stimulating hormone (TSH) receptor in thymocytes might be responsible for the autoimmune-mediated expansion of the thymus in Graves' disease due to the TSH receptor-stimulating autoantibodies [27, 28]. In addition to the previously described findings, we found that TRAD was associated with the Osserman type and age in MG patients, which was more common in patients with Osserman type I and younger patients. Previous studies have reported that patients positive for thyroxine antibodies were more likely to demonstrate an ocular MG phenotype and that ocular MG (particularly without thymus involvement or positive AchRAb) was associated with autoimmune thyroid diseases [29], consistent with our findings.

The limitations of this study are as follows. First, we had a small sample size of 33 patients, which could have led to biased results. Second, the study was retrospective, making it subject to bias, such as a more detailed evaluation of MG and ADs. In addition, because of the number of cases, the ADs in this study were relatively one-fold, and we expect a richer inclusion of ADs to facilitate more extensive research. However, conducting a prospective clinical study on MG complicated by AD is difficult as these diseases rarely occur in the population. We anticipate to conducting multicenter and multidisciplinary prospective studies in the future to provide more in-depth knowledge in this area and meaningful recommendations for clinical diagnosis and treatment.

## Conclusions

In conclusion, we conducted a study on the outcomes of patients with MG and AD synchronously after surgery. Long-term follow-up showed satisfactory improvement for both MG and ADs (more than 80% and nearly 70%). We found that the longer the time after the operation, the better the relief of MG. In patients with thymoma, the larger the diameter of the tumor, the better is the postoperative MG improvement. Among the enrolled patients with thymus, those with hyperplasia were predominantly female and young. The most common comorbid autoimmune disorder was TRAD, which was associated with thymic hyperplasia, Osserman type I MG, and young age.

## Data Availability

The datasets used and/or analyzed during the current study are available from the corresponding author on reasonable request.
